# The clinical value, regulatory mechanisms, and gene network of the cancer-testis gene *STK31* in pancreatic cancer

**DOI:** 10.18632/oncotarget.16814

**Published:** 2017-04-04

**Authors:** Kai Zhang, Zipeng Lu, Yi Zhu, Lei Tian, Jingjing Zhang, Chunhua Xi, Wentao Gao, Kuirong Jiang, Yi Miao

**Affiliations:** ^1^ Pancreatic Center & Department of General Surgery, The First Affiliated Hospital of Nanjing Medical University, Nanjing 210029, Jiangsu, China; ^2^ Pancreas Institute of Nanjing Medical University, Nanjing 210029, Jiangsu, China

**Keywords:** cancer-testis gene, pancreatic cancer, *STK31*

## Abstract

We aimed to identify *STK31* as a cancer-testis (CT) gene and to explore its potential clinical value, regulatory mechanisms, and gene network in pancreatic cancer (PC). Gene expression data were generated from normal organ samples and pancreatic cancer samples from three public databases. *STK31* expression patterns in normal and PC tissues were identified, and we explored its regulatory mechanisms. Gene ontology (GO) and pathway analyses of *STK31*-related genes were performed and an STK31 protein–protein interaction (PPI) network was constructed. *STK31* was confirmed as a CT gene in PC and its expression was significantly higher in patients with new neoplasm compared with patients without new neoplasm (*P* = 0.046) and in more advanced pathologic stages than in earlier stages (*P* = 0.002); methylation level correlated negatively with *STK31* expression. In total, 757 *STK31*-related genes were identified, and were significantly enriched in terms of polymorphisms and alternative splicings. The PPI network predicted that STK31 was physically associated with the PIWI (originally P-element Induced WImpy testis in *Drosophila*) and Tudor families.

## INTRODUCTION

PC is a highly malignant digestive tract disease with difficult early diagnosis and treatment. In almost 90% of patients, it originates from the epithelial gland ductal carcinoma [1, 2]. In the US, the five-year survival rate of PC remains as low as 6% [3]. The low survival rate is attributed to several factors, perhaps the most important of which is the late stage and metastasis when most patients are diagnosed [1–3]. Unfortunately, most patients are asymptomatic until it develops to an advanced stage.

The most well-established risk factor for PC is cigarette smoking, [4]. Chronic pancreatitis [5], diabetes persisting more than 20 years [6], high body mass index (BMI), and centralized fat distribution [7]. Previous studies have suggested four major driver genes of PC: *KRAS* (Kras proto-oncogene, GTPase), *CDKN2A* (cyclin-dependent kinase inhibitor 2A), *TP53* (tumor protein p53), and *SMAD4* (SMAD family member 4). These four genes are referred to as mutation driver genes of PC [3, 8]. However, these genes can only explain parts of pancreatic tumorigenesis; these genetic mutations are not present in many other patients with PC. Therefore, epigenetic drivers were put forward. Epigenetic drivers mean that epigenetic changes could alter gene expression, leading to the occurrence of tumors. They are now acknowledged as a universal feature of tumorigenesis [8]. Cancer-testis (CT) genes, whose expression is restricted to germ cells and is often reactivated and aberrantly expressed in cancers, are a group of epigenetic driver genes [9, 10]. Recently, patient-derived xenograft models of pancreatic ductal adenocarcinoma (PDAC) showed that JQ1, an inhibitor of CT genes in the bromodomain and extraterminal (BET) protein family (*BRDT*), suppresses PDAC development by inhibiting both MYC (v-myc avian myelocytomatosis viral oncogene homolog) activity and inflammatory signals [11]. This provided new insight into the molecular targets of PC. These findings all suggested that CT genes might play an important role in molecular targeted therapy of PC.

Recently, we found that *STK31* (serine/threonine kinase 31, also known as *TDRD8*) might be a novel CT gene in PC [10]. As a Tudor family member, *STK31* contains an STK domain and a Tudor domain, and participates in cell cycle regulation [12]. In mice, the homologous protein of STK31 is restricted to germ cells [13, 14] and is highly expressed in spermatogonia meiosis [13, 15, 16]. Moreover, *STK31* has been detected in colorectal cancer and is activated by demethylation [14]. In Caco2 and SW1116 colorectal cancer cells, *STK31* knockdown enhanced cell differentiation capacity, indicating that *STK31* maintains low differentiation in colorectal cancer cells [12, 14, 16].

In the present study, we deciphered the expression pattern of *STK31* and attempted to confirm whether it will be a good biomarker aiding clinical diagnosis and prognosis of PC. We also attempted to uncover the regulatory mechanisms and gene network of *STK31* in PC.

## RESULTS

### Tissue expression patterns and role of *STK31* in PC

**Figure 1:**
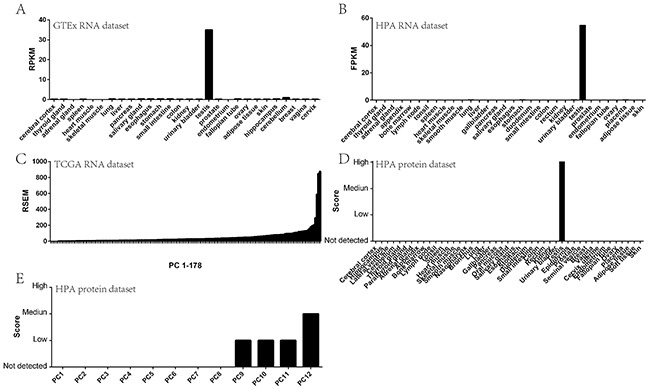
*STK31* tissue expression pattern *STK31* RNA expression in normal tissues from GTEx **(A)** and HPA **(B)**; *STK31* RNA expression in pancreatic cancer tissues from TCGA **(C)**; STK31 protein expression in normal tissues from HPA **(D)**; STK31 protein expression in pancreatic cancer tissues from HPA **(E)**. RPKM: Reads Per Kilobases per Millionreads; FPKM: Fragments Per Kilobase Million;

To determine whether *STK31* could be assigned to the CT genes expressed in PC, we first evaluated its expression pattern in normal human tissues including pancreas using transcriptomic data deposited in the Genotype-Tissue Expression Project (GTEx). *STK31* was mainly expressed in the testis (Figure 1A). The Human Protein Atlas (HPA) result was generally consistent with the GTEx data, showing that *STK31* was only expressed in the testis at both RNA and protein level (Figure 1B & 1D). Next, we evaluated *STK31* expression in PC specimens through bioinformatics analysis of RNA sequencing (RNA-seq) of The Cancer Genome Atlas (TCGA) PAAD data (178 PC samples), which indicated that *STK31* was elevated in about 85% of patients with PC (Figure 1C), which was also supported by the HPA (Figure 1E). These results confirm that *STK31* is a CT gene in PC.

**Figure 2:**
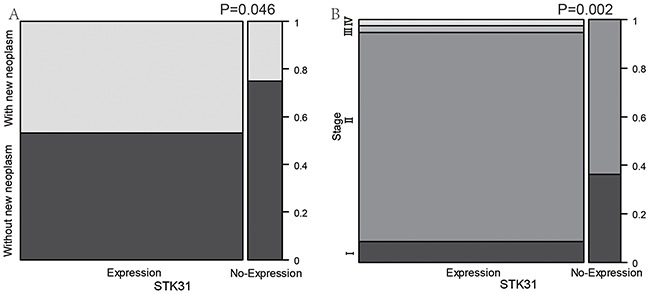
The association between *STK31* expression and clinical features of pancreatic cancer *STK31* expression was significantly higher in patients with new neoplasm compared patients without new neoplasm **(A)** and in more advanced pathologic stages than in earlier stages **(B)**.

**Figure 3:**
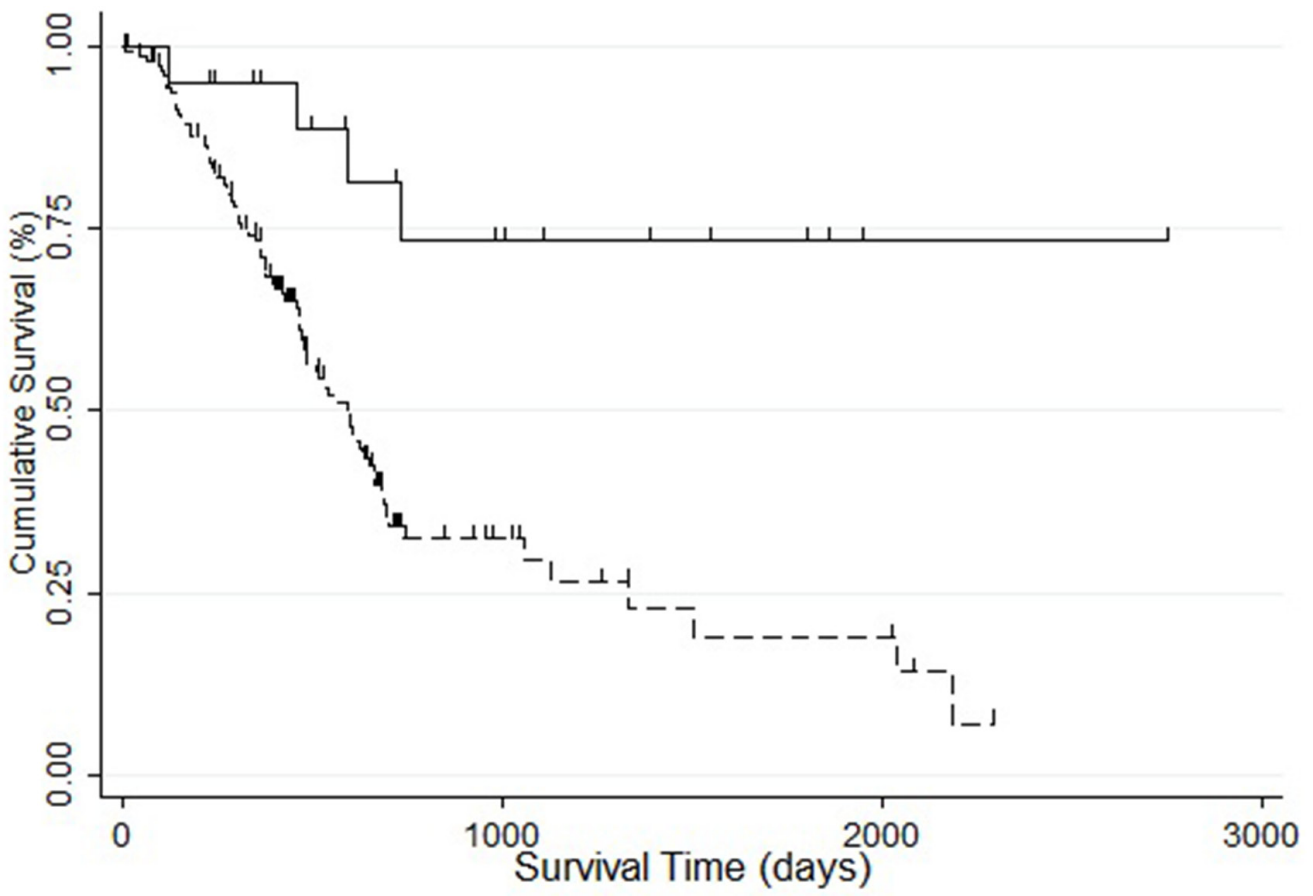
The association between *STK31* expression and survival of patients with pancreatic cancer Patients with *STK31* expression (RSEM>5, dotted line) had poorer survival than patients without *STK31* expression (RSEM≤5, solid line).

Interestingly, *STK31* expression was significantly higher in patients with new neoplasm compared with patients without new neoplasm (*P* = 0.046, Figure 2A). We also found that patients at more advanced pathologic stages tended to express *STK31* (*P* = 0.002, Figure 2B). To explore the association of *STK31* expression and the survival time of patients with PC, Kaplan–Meier survival curves based on *STK31* expression were constructed, showing that patients who expressed *STK31* had poorer survival (log-rank: *P* = 0.0009, Figure 3).

### The relationship between methylation, mutation, and *STK31* expression

**Figure 4:**
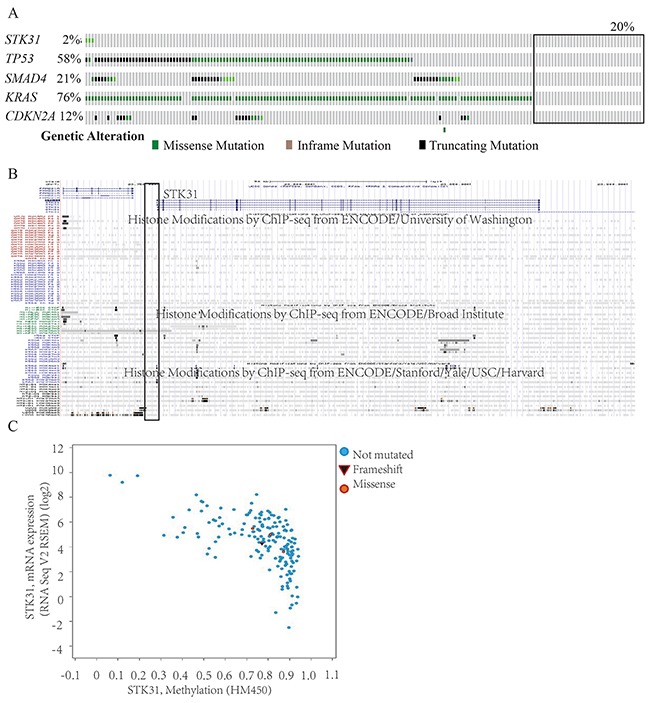
The relationship between methylation, mutation, and *STK31* expression **(A)** Only 2% of patients with pancreatic cancer carried *STK31* mutations; almost one-third of patients with pancreatic cancer (black rectangle) did not carry the four major mutation driver genes (*KRAS*, *CDKN2A*, *TP53*, *SMAD4*). **(B)** There was almost no histone modification in the *STK31* promoter region (2 kb upstream of *STK31*)). **(C)** Methylation levels (2 kb upstream of *STK31*) were negatively correlated with *STK31* expression.

In TCGA, almost one-fifth of patients with PC did not harbor the four major mutation driver genes in PC (*KRAS*, *CDKN2A*, *TP53*, *SMAD4*) (Figure 4A). Only 2% of patients carried *STK31* mutations. These results suggest another driving mode, such as epigenetic drivers, in PC. Further analysis showed that there was almost no histone modification in the *STK31* promoter region (2 kb upstream of the *STK31*) (http://genome.ucsc.edu/cgi-bin/hgGateway, Figure 4B). Interestingly, we found that methylation level (2 kb upstream of *STK31*) was negatively correlated with *STK31* expression (Pearson r=-0.634, Spearman r=-0.634, http://www.cbioportal.org/index.do, Figure 4C).

### The *STK31* gene network

**Figure 5:**
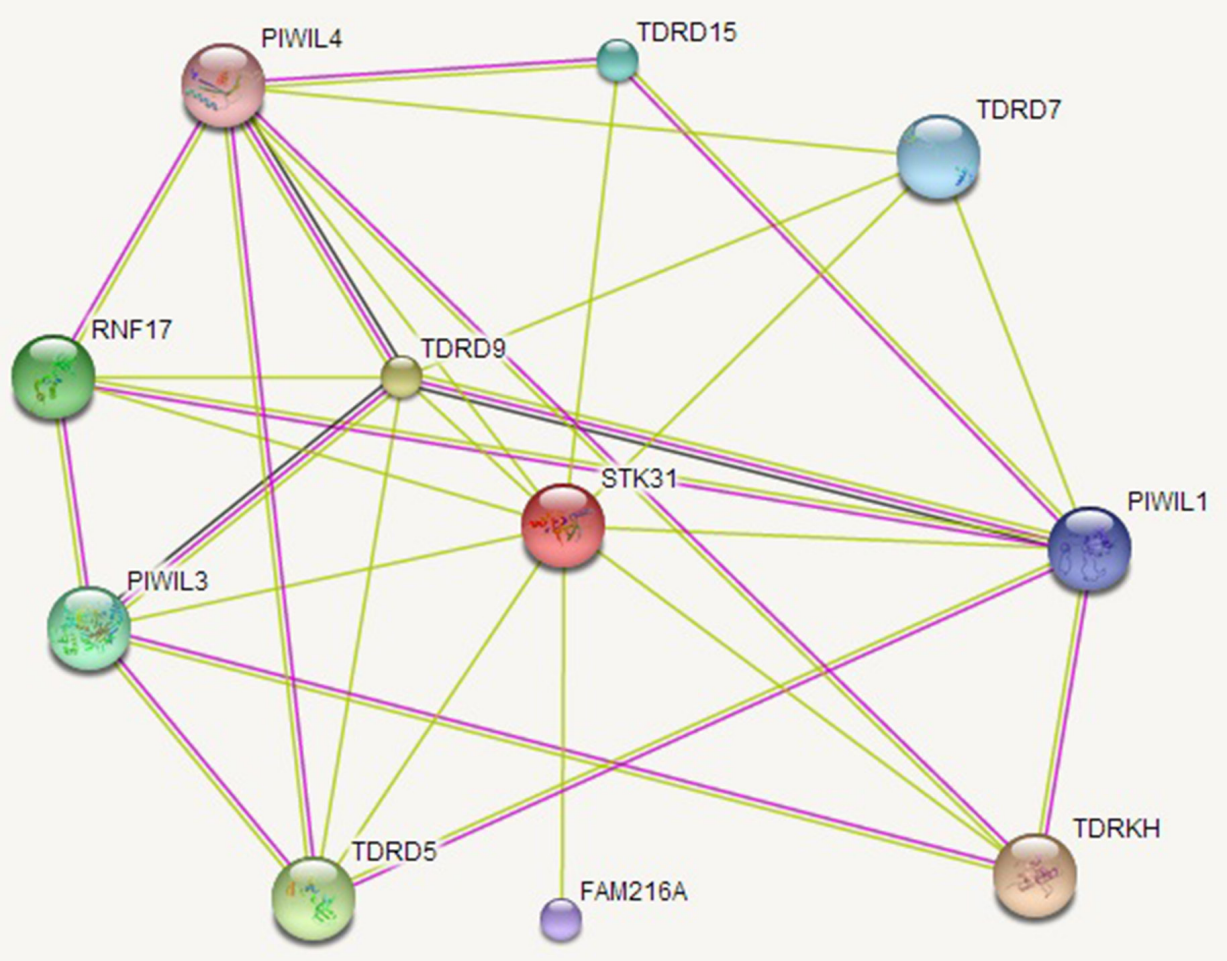
The *STK31* gene network *STK31* interacts with the PIWI subfamily of Argonaute proteins (*PIWIL1*, *PIWIL3*, *PIWIL4*) and the Tudor family (*TDRD5*, *TDRD7*, *TDRD9*, *TDRD15*, *TDRKH*).

We analyzed the relationship between *STK31* and 20,530 other genes. Seven hundred and fifty-seven genes were related with *STK31* (*P* < 1 × 10^-6^), where *STK31* was strongly associated with piwi-like RNA-mediated gene silencing 1 (*PIWIL1*) (r = 0.50, *P* = 5.56 × 10^-15^, Supplementary Table 1). A total 487 genes over-represented KEYWORDS terms related to polymorphism (false discovery rate, FDR: *P* < 0.001), representing genomic instability; only four Kyoto Encyclopedia of Genes and Genomes (KEGG) pathways (Table 1), including tight junction, adherens junction, glycosphingolipid biosynthesis, and axon guidance, were significantly enriched (FDR: *P* = 0.006, 0.006, 0.023, and 0.036, respectively) according to the Database for Annotation Visualization and Integrated Discovery (DAVID) (Supplementary Table 1). The Search Tool for the Retrieval of Interacting Genes (STRING 10.0) predicted physical interaction between *STK31* and the PIWI subfamily of Argonaute proteins (*PIWIL1*, *PIWIL3*, *PIWIL4*) and the Tudor family (*TDRD5*, *TDRD7*, *TDRD9*, *TDRD15*, *TDRKH*) (Figure 5).

**Table 1:** GO and pathway enrichment analysis of *STK31*-related genes

Category	Term	Count	FDR P-value
Keywords	Polymorphism	487	3.10E-05
Keywords	Alternative splicing	442	6.20E-06
Keywords	Phosphoprotein	343	6.50E-08
Keywords	Membrane	289	2.40E-02
Keywords	Cytoplasm	231	2.10E-06
Keywords	Nucleotide-binding	91	4.90E-03
Keywords	Transferase	89	7.80E-04
Keywords	Ubl conjugation	87	4.30E-03
Keywords	ATP-binding	79	6.00E-04
Keywords	Cytoskeleton	72	3.60E-05
Keywords	Cell cycle	58	1.40E-08
Keywords	Developmental protein	55	5.20E-03
Keywords	Cell division	46	1.80E-10
Keywords	Cell junction	44	1.40E-03
Keywords	Kinase	41	3.20E-02
Keywords	Mitosis	39	2.30E-11
Keywords	Apoptosis	36	5.90E-03
Keywords	Chromosome	32	3.30E-04
Keywords	Glycosyltransferase	25	9.70E-05
Keywords	Centromere	24	1.70E-08
Keywords	SH3 domain	23	2.40E-04
Keywords	Microtubule	23	6.80E-03
Keywords	Kinetochore	20	2.50E-08
Keywords	Motor protein	15	5.00E-03
Keywords	Tight junction	14	2.30E-04
Keywords	Tyrosine protein kinase	13	8.60E-03
Keywords	Microsome	13	2.60E-02
Keywords	Chromosome partition	10	4.40E-04
Keywords	Epidermolysis bullosa	7	2.20E-04
Keywords	Basement membrane	7	3.20E-02
GO analysis			
MF	Protein kinase binding	32	4.90E-02
CC	Cytoplasm	239	2.60E-05
CC	Cytosol	176	9.00E-06
CC	Extracellular exosome	150	9.90E-05
CC	Nucleoplasm	129	1.00E-02
CC	Focal adhesion	30	1.20E-02
CC	Midbody	21	1.30E-05
CC	Cell–cell junction	17	1.10E-02
CC	Kinetochore	16	1.20E-05
CC	Condensed chromosome kinetochore	16	1.50E-05
CC	Bicellular tight junction	16	9.60E-04
CC	Chromosome, centromeric region	13	2.50E-05
CC	Spindle pole	12	2.60E-02
CC	Spindle microtubule	10	1.40E-03
CC	Brush border	10	3.00E-03
CC	Mitotic spindle	8	1.60E-02
CC	Desmosome	7	7.00E-03
CC	Hemidesmosome	5	4.50E-03
BP	Small GTPase–mediated signal transduction	49	1.50E-02
BP	Apoptotic process	41	4.00E-02
BP	Cell division	37	6.60E-06
BP	Mitotic cell cycle	36	5.90E-03
BP	Mitotic nuclear division	32	3.50E-06
BP	Cell proliferation	32	6.80E-03
BP	Cell migration	20	6.60E-03
BP	Cell junction assembly	19	1.30E-06
BP	Ephrin receptor signaling pathway	17	2.20E-04
BP	Chromosome segregation	16	4.70E-05
BP	Anterior/posterior pattern specification	13	1.60E-02
BP	Single organismal cell–cell adhesion	13	3.00E-02
BP	Epidermis development	12	3.50E-02
BP	Cell–cell junction organization	11	2.90E-02
BP	Bicellular tight junction assembly	10	6.10E-03
BP	O-glycan processing	10	3.60E-02
BP	Hemidesmosome assembly	7	9.10E-04
BP	Mitotic sister chromatid segregation	7	2.10E-02
BP	Phosphatidylethanolamine acyl-chain remodeling	7	2.60E-02
BP	Mitotic cytokinesis	7	4.60E-02
BP	Branching involved in mammary gland duct morphogenesis	6	1.60E-02
BP	Negative regulation of cellular glucuronidation	5	2.20E-02
BP	Negative regulation of glucuronosyltransferase activity	5	2.20E-02
BP	Negative regulation of fatty acid metabolic process	5	2.90E-02
BP	Xenobiotic glucuronidation	5	2.90E-02
pathway			
KEGG_PATHWAY	Tight junction	16	6.80E-03
KEGG_PATHWAY	Axon guidance	15	2.30E-02
KEGG_PATHWAY	Adherens junction	12	7.40E-03
KEGG_PATHWAY	Glycosphingolipid biosynthesis - lacto and neolacto series	7	3.00E-02

## DISCUSSION

*STK31* is highly conserved in humans, chimpanzees, and gorillas. Previous reports have stated that the STK domain is required for regulating cancer cell differentiation [14] and STK expression is often altered in human cancers [17].

The present study demonstrates that, except the testes, *STK31* is not expressed in normal tissues but aberrantly expressed in PC tissues, indicating that *STK31* is a CT gene in PC. Whether *STK31* is expressed is related to the pathologic stage, new neoplasm status, and prognosis of PC, which might be a new means of aiding clinical diagnosis and estimating the degree of severity. As lower *STK31* expression is beneficial for patients with PC, perhaps attempts should be made to inhibit the expression of *STK31*. These inhibitors of STK31 are akin to a molecular therapeutic target, to improve the survival of patients with PC. There are numerous commercially available inhibitors of STKs now including inhibitors of the *STK31* STK domain [18].

Currently, there are four major mutation driver genes of PC: *KRAS*, *CDKN2A*, *TP53*, and *SMAD4*. However, only about two-thirds of patients with PC carry one or more mutations on these genes. We also found that only 2% of patients with PC carry *STK31* mutations. Interestingly, we found that *STK31* was activated by demethylation, which is also an important mechanism for the reactivation of most CT genes [9, 19, 20], which is consistent to Yokoe's study [21].

In the testis, *STK31* expression is limited to spermatogonia [22], indicating its key role in germ cell differentiation. However, Fok and his group subsequently found that *STK31* could also regulate colon cancer cell differentiation [14]. In this present study, we found that *STK31* interacts with the PIWI subfamily (*PIWIL1*, *PIWIL3*, *PIWIL4*), which is confirmed by co-immunoprecipitation assay *in vivo* and *in vitro* in mice testes [12]. PIWI subfamily comprised of evolutionarily conserved proteins containing both PAZ and Piwi motifs. It plays an important role in stem cell self-renewal, RNA silencing, and translational regulation. And *STK31* also interacts with the Tudor family (*TDRD5*, *TDRD7*, *TDRD9*, *TDRD15*, *TDRKH*), an evolutionarily conserved family of proteins involved in germ cell development. These two families have long been interrelated. *PIWIL1*, *PIWIL3*, and *PIWIL4* act as intrinsic regulators of the self-renewal capacity of germ line and hematopoietic stem cells, and are believed to be involved in cancer development [23–26]. *TDRD5*, *TDRD9*, and *TDRKH* are essential for PIWI-interacting RNA (piRNA)-mediated retrotransposon silencing in the male germline [27–29].

In conclusion, *STK31* is a CT gene and is reactivated by demethylation. *STK31* expression is significantly higher in relapsed patients, or patients with advanced pathologic stage or poorer prognosis, suggesting that *STK31* might be of potential clinical value. *STK31* interacts with the PIWI and Tudor families, which suggests that it might play a key role in maintaining genomic instability. Molecular targeting treatment has evolved along with better understanding of the mechanisms of cancer, and *STK31* may be a good molecular therapeutic target in PC.

## MATERIALS AND METHODS

### Public datasets

We used multiple public databases containing data on both normal and PC tissues to evaluate the expression pattern of *STK31*. The GTEx contains information on gene expression in multiple normal tissues, including the pancreas and testis (http://www.gtexportal.org/home/) [30]. The HPA presents the expression levels of both RNA and protein in normal and tumor tissues (http://www.proteinatlas.org/) [31]. The transcriptional profile and clinical data of PC were obtained from PAAD datasets in TCGA (released on June 1, 2015 https://tcga-data.nci.nih.gov/tcga/tcgaHome2.jsp) [32]. In total, 178 samples had both gene expression and clinical data. Level 3 released gene expression data for RNA-seq was performed RNA-Seq by Expectation Maximization (RSEM). RSEM is an accurate transcript quantification from RNA-Seq data [33].

### *STK31* tissue expression pattern

*STK31* expression data were extracted from the above databases and the differential expression levels between clinical statuses were analyzed using the chi-square test. Survival analysis was used to evaluate the prognostic role of *STK31* in PC, and log-rank testing was used to determine the significance for Kaplan–Meier analyses to uncover the indication for survival time.

### *STK31* regulatory mechanism and gene ontology (GO) analysis

Correlation analysis was performed to establish a relationship between methylation and *STK31* expression, which suggested potential regulation of *STK31*. The role of histone modification in promoter region (2 kb upstream of the STK31) to the STK31 was assessed in UCSC genome browser [34]. The relationship of all other genes (20,531 genes) with *STK31* was assessed in the RNA-seq of TCGA PAAD data, which used the Spearman test and considered genes with Spearman *P* < 1 × 10^-6^ as *STK31*-related. The GO analysis was executed by DAVID 6.8 Beta [35], which systematically extracts biological pathways from large gene lists. The Functional_Categories (KEYWORDS) and pathway (KEGG pathway) of the *STK31*-related genes were analyzed using DAVID with FDR *P* < 0.05 (based on the hypergeometric distribution) and count ≥ 2 (number of genes).

### Protein–protein interactions (PPI) network analysis

The STRING 10.0 [36] database is commonly used to retrieve predicted protein interactions. STRING 10.0 covers a total 2031 organisms and 9,643,763 proteins. All PPI obtained by STRING 10.0 have confidence scores. We searched the *STK31*-interacting genes, and selected genes with a confidence score ≥ 0.4 to construct the PPI network.

## SUPPLEMENTARY MATERIALS TABLES




