# Supplementary material for: The clinical value, regulatory mechanisms, and gene network of the cancer-testis gene STK31 in pancreatic cancer
Source: Oncotarget. 2017 Apr 4;8(21):35154–64. doi: 10.18632/oncotarget.16814 (PMC5471042; doi:10.18632/oncotarget.16814)
Supplement: Supplementary file 1 [file oncotarget-08-35154-s001.pdf]

The clinical value, regulatory mechanisms, and gene network of the cancer-testis gene *STK31* in pancreatic cancer

SUPPLEMENTARY TABLE

Supplementary Table 1: Go analysis for these related genes of STK31.

See Supplementary File 1
